# Evaluation of fecal DNA extraction protocols for human gut microbiome studies

**DOI:** 10.1186/s12866-020-01894-5

**Published:** 2020-07-17

**Authors:** Mi Young Lim, Yong-Soo Park, Jung-Ha Kim, Young-Do Nam

**Affiliations:** 1 Research Group of Healthcare, Korea Food Research Institute, Jeollabuk-do, 55365 Republic of Korea; 2 Food Processing Support Team, Korea Food Research Institute, Jeollabuk-do, 55365 Republic of Korea; 3 Department of Family Medicine, Chung-Ang University Hospital, Chung-Ang University College of Medicine, Seoul, 06973 Republic of Korea; 4 Department of Food Biotechnology, Korea University of Science and Technology, Daejeon, 34113 Republic of Korea

**Keywords:** DNA extraction, Gut microbiome, 16S rRNA gene sequencing, Fecal sample

## Abstract

**Background:**

DNA extraction is an important factor influencing the microbiome profile in fecal samples. Considering that the QIAamp DNA Stool Mini Kit, one of the most commonly used DNA extraction kits, is no longer manufactured, this study aimed to investigate whether a new commercially available kit, the QIAamp PowerFecal Pro DNA Kit, yields comparable microbiome profiles with those previously obtained using the QIAamp DNA Stool Mini Kit.

**Results:**

We extracted DNA from fecal samples of 10 individuals using three protocols (protocol P of the QIAamp PowerFecal Pro DNA Kit, and protocols SB and S of the QIAamp DNA Stool Mini Kit with and without an additional bead-beating step, respectively) in triplicate. Ninety extracted DNA samples were subjected to 16S rRNA gene sequencing. DNA quality measured by 260/280 absorbance ratios was found to be optimal in protocol P. Additionally, the DNA quantity and microbiome diversity obtained using protocol P were significantly higher than those of protocol S, however, did not differ significantly from those of protocol SB. Based on the overall microbiome profiles, variations between protocol P and protocol SB or S were significantly less than between-individual variations. Furthermore, most genera were not differentially abundant in protocol P compared to the other protocols, and the number of differentially abundant genera, as well as the degree of fold-changes were smaller between protocols P and SB than between protocols P and S.

**Conclusions:**

The QIAamp PowerFecal Pro DNA Kit exhibited microbiome analysis results that were comparable with those of the QIAamp DNA Stool Mini Kit with a bead-beating step. These results will prove useful for researchers investigating the gut microbiome in selecting an alternative protocol to the widely used but discontinued kit.

## Background

Studies on the human gut microbiome have increasingly reported associations between the gut microbiome and various diseases including type 2 diabetes [1], or inflammatory bowel disease [2]. Generally, gut microbiome studies are performed with experimental methods that differ among research groups, resulting in studies that have technical variations, thereby hindering the capacity to draw direct comparisons between multiple studies.

Experimental steps including sample preservation [3, 4], DNA extraction [5–7], and library preparation [8] influence microbiome profiles. Among these, DNA extraction from fecal samples is a major factor affecting the variability of the microbiome profiles according to the International Human Microbiome Standards (IHMS) group [9], the MicroBiome Quality Control (MBQC) project [10], among other studies [11, 12]. Despite the use of the same DNA extraction kit, the inclusion of a mechanical lysis step can also affect the resulting microbial composition as a higher mechanical strength is needed to disrupt the cell walls of gram-positive bacteria [5, 6, 9].

The QIAamp DNA Stool Mini Kit was one of the most commonly used extraction kits for gut microbiome studies. The IHMS reported that the protocol for this kit, with specific modifications, displays optimal performance in overall microbiome analysis [9]. However, the manufacturing of this kit has been discontinued; hence, numerous research groups may now require a new DNA extraction protocol. As an alternative, the QIAamp PowerFecal Pro DNA Kit, recently manufactured by Qiagen, may prove useful owing to its improved technology for the efficient elimination of inhibitors in fecal samples, as well as the application of a novel bead tube to facilitate more efficient bacterial lysis [13]. Furthermore, this kit is compatible with an existing automated sample preparation instrument, QIAcube, and thus, the downstream processes following bead-beating can be automated using the QIAcube. The use of automated systems for DNA extraction allows for the elimination of manual experimental steps, thereby, enabling the standardization of results. Before introducing a new protocol for the primary experiments, it is important, however, to determine whether the new protocol would be an appropriate replacement for gut microbiome studies in comparison with the established kits and protocols.

This study investigated whether the QIAamp PowerFecal Pro DNA Kit (protocol P) performed similarly to the QIAamp DNA Stool Mini Kit with or without an additional bead-beating step (protocols SB and S, respectively) in gut microbiome analysis. To this end, fecal samples obtained from 10 individuals were collected. The similarities or differences among the three protocols were then assessed by 16S rRNA gene sequence profiles, as well as the quantity and quality of extracted DNA. This study provides useful information regarding the differences in human gut microbiome data generated using three protocols and will, therefore, prove valuable for researchers in selecting alternative protocols for investigating the gut microbiome.

## Results

### DNA quantity and quality

**Fig. 1:**
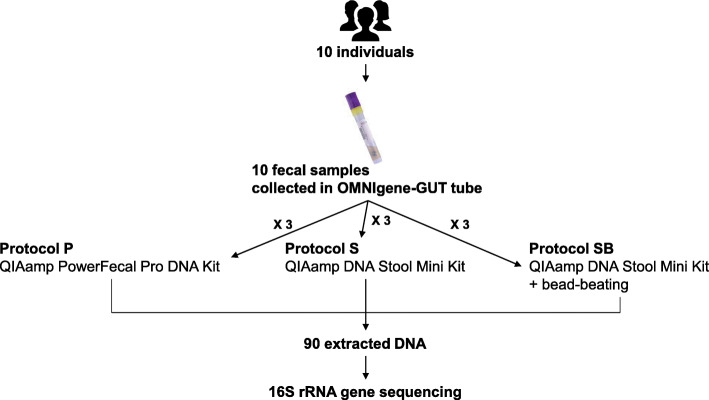
Experimental design. Ten fecal samples mixed with OMNIgene-GUT solution were used for DNA extraction with three DNA extraction protocols in triplicates. Ninety DNA samples thus extracted were subjected to 16S rRNA sequencing

**Fig. 2:**
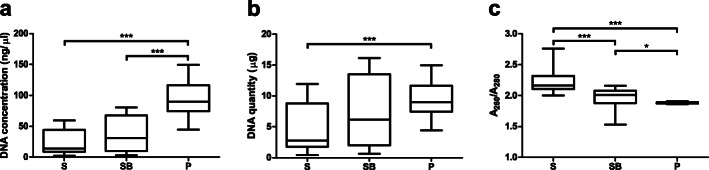
Concentration and purity of the DNA samples. **a** Concentration, **b** quantity, and **c** purity (A_260_/A_280_) of the DNA samples extracted through protocols S, SB, and P. (* *P*-value < 0.05, *** *P*-value < 0.001; Kruskal-Wallis test with Dunn’s multiple comparison test)

Ninety DNA samples were extracted from 10 individual fecal samples using three protocols in triplicate aliquots (three protocols × 10 individuals × triplicate) (Fig. 1). One sample was excluded from all downstream analyses as it appeared to be mislabeled or to have a technical error, based on the 16S rRNA sequence results. To assess whether the quantity and quality of extracted DNA were appropriate for library preparation for 16S rRNA gene sequencing, we first measured the DNA concentration and DNA purity using a NanoDrop ND-1000 spectrophotometer. DNA concentration was the highest for protocol P (*P*-value < 0.0001; mean ± SD: protocol P 93.97 ± 27.73 ng/μL, protocol SB 35.84 ± 27.46 ng/μL, protocol S 23.74 ± 18.33 ng/μL; Fig. 2a). Considering the elution volumes, DNA quantities of 9.397 ± 2.773 μg, 7.168 ± 5.491 μg, 4.748 ± 3.666 μg were obtained per extraction with protocols P, SB, and S, respectively (Fig. 2b). Furthermore, samples obtained using protocol P had 260/280 absorbance ratios closest to 1.8, indicating that these samples were the least contaminated with proteins or RNAs (*P*-value < 0.0001; mean ± SD: protocol P 1.884 ± 0.0138, protocol SB 1.962 ± 0.1693, protocol S 2.234 ± 0.1886; Fig. 2c). Moreover, protocol P displayed the lowest standard deviation for DNA quantity and quality (Fig. 2), indicating that protocol P yielded DNA with consistent quantity and quality from fecal samples. Additionally, although the 260/230 absorbance ratios in protocol P were lower than the desirable range (2.0–2.2) (Additional file 1: Fig. S1), which may have been affected by residual buffer components from the extraction steps, the 16S libraries for all samples were well constructed. This indicates that all tested protocols effectively extracted DNA of sufficient quantity and quality to construct the 16S libraries.

### Microbial diversity

**Fig. 3:**
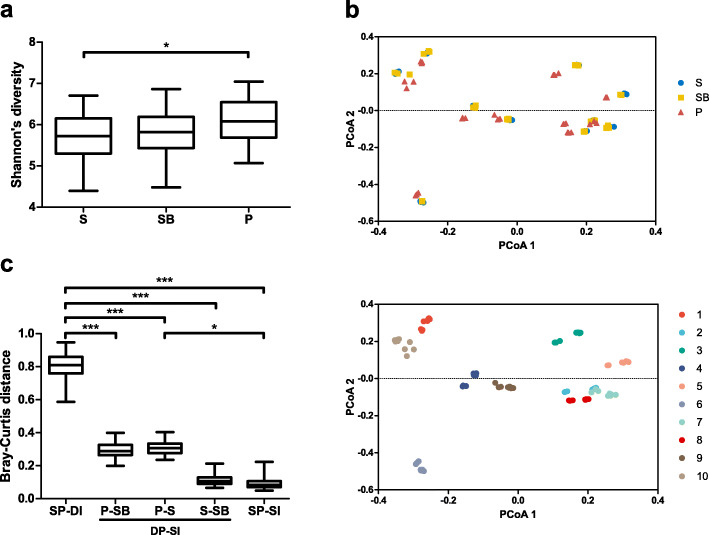
Diversity metrics of the samples. **a** Alpha diversity (Shannon’s diversity). **b** Principal coordinates analysis based on Bray-Curtis distances. The samples were color-coded for the DNA extraction protocols (top) and for individuals (bottom), respectively. **c** Bray-Curtis distances 1) between samples from the same protocol and different individuals (SP-DI), 2) between samples from different protocols and the same individual (DP-SI: P vs SB, P vs S, and S vs SB), and 3) between samples from the same protocol and the same individual (SP-SI, replicate samples). (* *P*-value < 0.05, *** *P*-value < 0.001; Kruskal-Wallis test with Dunn’s multiple comparison test)

Further, we analyzed the effect of DNA extraction protocols on the gut microbiome data through 16S rRNA sequencing with the Illumina MiSeq platform. Costea et al. reported that the alpha diversity index (Shannon’s diversity) serves as an optimal criterion for DNA extraction performance as it displays positive correlations with the recovered relative abundance of gram-positive bacteria [9]. We thus compared Shannon’s diversity index for the three protocols to predict the accuracy of the recovered abundance profile. Significant differences in the Shannon’s diversity were observed exclusively between protocols S and P (*P*-value < 0.05), with no differences observed between protocols SB and P (Fig. 3a). Therefore, based on Shannon’s diversity index, protocol P appeared to offer the optimal performance, albeit comparable to that of protocol SB.

We also compared the microbial richness and evenness and found that the mean values of observed amplicon sequence variants (ASVs) were highest in the protocol P samples, however, these differences were not statistically significant across protocols. Meanwhile, the Pielou’s evenness of protocol P was significantly higher than for protocols S and SB (Additional file 2: Fig. S2), indicating that although the extraction protocols did not exhibit differences in the number of distinct microbes, they did affect the relative abundances of observed microbes. These differences in the relative abundances may lead to microbial evenness as well as Shannon’s diversity.

### Variation in microbiome profiles

To evaluate the effect sizes of DNA extraction protocols on gut microbiome profiles, the beta diversity was determined in accordance with the Bray-Curtis distance. The principal coordinates analysis (PCoA) plot revealed that samples from each individual clustered together irrespective of the extraction protocol (Fig. 3b).

Furthermore, we determined the Bray-Curtis distances, 1) between samples from the same protocol and different individuals, 2) between samples from different protocols and the same individual (P vs SB, P vs S, and S vs SB), and 3) between samples from the same protocol and the same individual (replicate samples) (Fig. 3c). As shown in the PCoA plot (Fig. 3b), the samples from the same protocol and different individuals displayed significantly greater Bray-Curtis distances (mean ± SD: 0.8063 ± 0.07258) compared to those between P vs SB, P vs S, and S vs SB or those between replicate samples, indicating that inter-individual variation is greater than inter-protocol or intra-protocol variations. Furthermore, Bray-Curtis distances of P vs SB and P vs S were slightly, however not significantly, higher than those of S vs SB (mean ± SD: P vs SB 0.2945 ± 0.04676, P vs S 0.3090 ± 0.04252, S vs SB 0.1138 ± 0.03161). Together, these results indicate that inter-individual differences largely influenced the gut microbiome profiles compared to the inter-protocol differences, and that individual microbial profiles generated with the different protocols were generally comparable.

### Differences in genus abundance

**Fig. 4:**
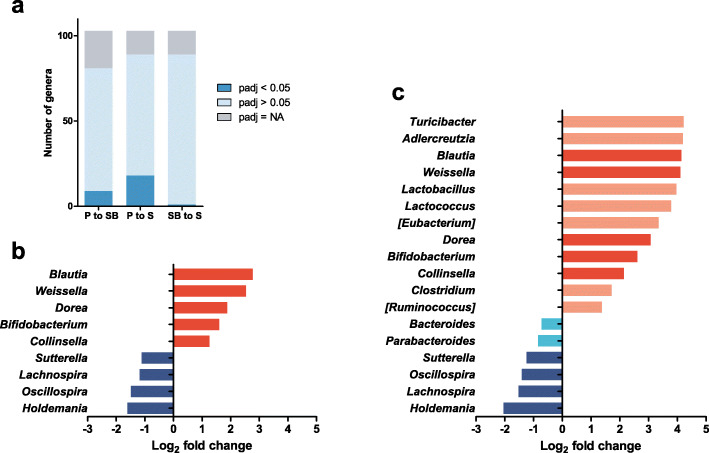
DESeq2 analysis identifying differentially abundant genera between samples obtained through protocols S, SB, and P. **a** The numbers of significantly differentially abundant genera (padj < 0.05), and non-significantly differentially abundant genera (padj > 0.05) among the 103 tested genera. **b** and **c** Genera to the right and the left of the zero line are more and less abundant in protocol P than in protocols SB **b** or S **c**, respectively. Brighter colored bars indicate that the genera were differentially abundant in protocol P in comparison only with protocol S, but not with protocol SB. Darker colored bars indicate that the genera were commonly differentially abundant in protocol P compared to both protocols SB and S

We next performed differential abundance analysis to enumerate and determine the significantly differentially abundant genera between the samples obtained using each protocol. During this analysis, if all samples were assigned a zero count for a given genus, or if a genus was filtered by automatic independent filtering, the adjusted *P*-value (padj) was set to NA for the genus in DESeq2. Accordingly, among the 103 taxa classified at the genus level, the adjusted *P*-values were not applicable for 22 (21.4%) genera for the comparison between protocols P and SB and for 14 (13.6%) genera in comparisons both between protocols P and S, and between protocols SB and S. Moreover, 72 (69.9%) and 71 (68.9%) genera were not differentially abundant in protocol P compared with protocols SB and S, respectively; meanwhile, 88 (85.4%) genera were not differentially abundant in protocol SB compared to protocol S (padj > 0.05). Alternatively, nine (8.7%) and 18 (17.5%) of the tested genera were differentially abundant in protocol P compared to protocols SB and S, respectively; while only one genus (1.0%) was found to be differentially abundant in protocol SB compared to protocol S (padj < 0.05; Fig. 4a and Additional file 3: Table S1).

Samples from protocol P had significantly higher abundances of *Blautia*, *Weissella*, *Dorea*, *Bifidobacterium*, and *Collinsella*, while these samples had significantly lower abundances of *Holdemania*, *Oscillospira*, *Lachnospira*, and *Sutterella* than those from both protocols SB and S (Figs. 4b and c). The degrees of log_2_ fold-changes for these genera were higher between protocols P and S than between protocols P and SB, indicating that the abundances of these genera were affected by differences between the two kits, and the magnitude of the effect increased upon omission of an additional bead-beating step. These patterns were also observed in the graphs for genera relative abundance among the three protocols (Additional file 4: Fig. S3, Additional file 5: Fig. S4).

Compared to the samples obtained using protocol S, those obtained with protocol P additionally exhibited a higher abundance of other genera including *Turicibacter*, *Adlercreutzia*, *Lactobacillus*, *Lactococcus*, [*Eubacterium*], *Clostridium*, and [*Ruminococcus*], while having a lower abundance of *Bacteroides* and *Parabacteroides* (Figs. 4b and c). Interestingly, the genera determined to be more abundant in protocol P than in protocol S, but not than in protocol SB were gram-positive bacteria, while those that were less abundant in protocol P than in protocol S, but not than in protocol SB were gram-negative bacteria. These results are concurrent with the reduction in the alpha diversity in protocol S compared to protocol P (Fig. 3a), thus reconfirming that inclusion of a bead-beating step in the DNA extraction protocol is critical for disrupting the cell wall of gram-positive bacteria [5, 6].

Among the top 10 most abundant genera, differential abundance analysis of protocols P and SB revealed significant differences only in the 9th and 10th most abundant genera (*Oscillospira* and *Blautia,* respectively). In contrast, on comparing protocols P and S, four genera, including the most abundant (*Bacteroides*), were found to be differentially abundant between the two protocols among the top 10 most abundant genera (Additional file 3: Table S1). Therefore, when compared to protocol SB, the effect of protocol P on the abundances of major genera was smaller than compared to protocol S.

## Discussion

This study shows that the degree of microbiome variations caused by differences in the tested DNA extraction protocols was significantly less than that caused by inter-individual differences. Meanwhile, the results of Shannon’s diversity and differential abundance analysis suggest that protocol P gives more similar results to protocol SB as compared to protocol S, which may be due to the fact that both protocols P and SB included a bead-beating step during DNA extraction, while protocol S did not. Hence, bead-beating as implemented in protocols P and SB provides higher extraction efficiency for gram-positive bacteria, concurrent with previous reports [6, 9, 14].

A limitation of this study was the exclusion of other commercially available kits for the comparative analysis. It is important to maintain consistent protocols throughout a microbiome study; however, in the case of a long-term study that carries over several years, a product can become discontinued. The study presented herein proves particularly important in these instances to provide a clear comparison of the similarities and differences in microbiome profiles generated between the discontinued kit and an alternative. We note that in this context, the current study focused on a comparison between the widely used but discontinued kit (QIAamp DNA Stool Mini Kit) and a newly available kit (QIAamp PowerFecal Pro DNA Kit), rather than a comparison between the new kit and other commercially available kits, or a comparison between the discontinued kit and other commercially available kits. To determine which protocol is the most suitable for gut microbiome study among the commercially available kits, future studies with a larger number of kits are needed.

Despite the limitation, this study provides useful information for many researchers who had previously used the QIAamp DNA Stool Mini Kit, or who will analyze the microbiome data generated with QIAamp DNA Stool Mini Kit and QIAamp PowerFecal Pro DNA Kit, as well as for those who are preparing to set up a new microbiome experiment.

## Conclusions

This study shows that protocol P of the new commercially available DNA extraction kit (QIAamp PowerFecal Pro DNA Kit) yields generally comparable microbiome results with those from the existing protocol including a bead-beating step, with only few genera being differentially abundant. The present results are potentially applicable to researchers for the selection process of an alternative DNA extraction protocol in place of the discontinued kit (QIAamp DNA Stool Mini Kit). Owing to differences in the abundance of several bacteria among different extraction protocols, when comparing multiple studies, the extraction protocols used in the studies must be considered.

## Methods

### Fecal samples and DNA extraction

Ten healthy fecal samples collected in OMNIgene-GUT tubes (DNA Genotek, ON, Canada) were used for DNA extraction experiments. Herein, two commercial DNA extraction kits were used: QIAamp DNA Stool Mini Kit (Qiagen, Hilden, Germany) and QIAamp PowerFecal Pro DNA Kit (Qiagen). For the QIAamp DNA Stool Mini Kit and QIAamp PowerFecal Pro DNA Kit, DNA extraction was performed with 250 μL of the fecal sample in accordance with the manufacturer’s instructions, using a QIAcube system (Qiagen), being designated as “protocol S” and “protocol P,” respectively. Furthermore, because the QIAamp DNA Stool Mini Kit does not include a bead-beating step, DNA extraction performed using this kit involved an additional bead-beating step, being designated as “protocol SB” [5]. Briefly, 250 μL of the fecal sample was transferred to a 2-mL tube containing 1.2 mL ASL lysis buffer and 0.3 g sterile 0.1 mm zirconia beads (BioSpec, OK, USA), and then vortex-mixed for 2 min. The samples were heated at 95 °C for 15 min and additionally homogenized using the Qiagen TissueLyser II. After treatment with an InhibitEX Tablet, 350 μL of the supernatant was subjected to the subsequent steps using a QIAcube system. In protocol P, DNA was eluted in 100 μl of elution buffer, while in protocols S and SB, DNA was eluted in 200 μl of elution buffer. For individual fecal samples, DNA was extracted with each protocol (S, SB, or P protocol) in triplicate, thus yielding 90 DNA samples for further analyses (three protocols × 10 individuals × triplicate) (Fig. 1). DNA quantity and quality (A_260_/A_280_ and A_260_/A_230_) were measured using a NanoDrop ND-1000 spectrophotometer (NanoDrop Technologies Inc., DE, USA) and the rest of the DNA was stored at − 20 °C until use.

### 16S rRNA sequencing and sequence processing

A library for the V3–V4 region of 16S rRNA was constructed in accordance with the 16S Metagenomic Sequencing Library Preparation Illumina protocol (Part # 15044223 Rev. B, Illumina, CA, USA). Sequencing was performed using the MiSeq 2 × 300 platform (Illumina) in accordance with the manufacturer’s instructions.

From the sequencing results, ASVs were inferred using DADA2 plugin [15] within QIIME2 (2019.10 version) [16]. To predict the taxonomy of each ASV, the Naïve Bayesian classifier [17] was trained on the V3–V4 region of the Greengenes 13.8 database [18], and then applied to the ASV sequences. Alpha diversity (Shannon’s diversity, observed ASVs, and Pielou’s evenness) and beta diversity (Bray-Curtis distance) were determined using the q2-diversity plugin in QIIME2 at a sampling depth of 12,500.

### Statistical analysis

PCoA was performed for the Bray-Curtis distance metric using the q2-diversity plugin in QIIME2. The Kruskal-Wallis test with Dunn’s multiple comparison test was conducted to compare the variables among the three protocol groups. To identify differentially abundant genera between the samples obtained through protocols S, SB, and P, differential abundance analysis was performed using the ASV table agglomerated to the genus level, using a negative binomial Wald test as implemented in the DESeq2 R package [19]. We considered a Benjamini–Hochberg adjusted *P*-value < 0.05 as statistically significant. One of the individual #5 DNA samples extracted using the SB protocol (SB5–1) was excluded from all analyses, as based on the Bray-Curtis distances between replicate samples, the distances between SB5–1 and the other two SB5 (SB5–2 and SB5–3) were substantially greater, not only than those between other replicate samples, but also compared to distance between SB5–2 and SB5–3, which may be caused by a mislabeling or technical error.

## Supplementary information

**Additional file 1: Figure S1.** A_260_/A_230_ of the DNA samples extracted through protocols S, SB, and P. (** *P*-value < 0.01, *** *P*-value < 0.001; Kruskal-Wallis test with Dunn’s multiple comparison test).

**Additional file 2: Figure S2.** Alpha diversity (observed ASVs and Pielou’s evenness) of the samples extracted through protocols S, SB, and P. (* *P*-value < 0.05, ** *P*-value < 0.01; Kruskal-Wallis test with Dunn’s multiple comparison test).

**Additional file 3: Table S1.** DESeq2 analysis identifying differentially abundant genera between the samples obtained through protocols S, SB, and P.

**Additional file 4: Figure S3.** Average relative abundance plot for DNA extraction protocols (the top 20 most abundant genera).

**Additional file 5: Figure S4.** The relative abundances of several significantly differentially abundant genera obtained through protocol P in comparison with protocols SB and S.

## Data Availability

The datasets generated and/or analyzed during the current study are available the European Nucleotide Archive (ENA) under accession number PRJEB37658, http://www.ebi.ac.uk/ena/data/view/PRJEB37658.

## Abbreviations

PCoA: Principal coordinates analysis
Padj: Adjusted *P*-value
ASVs: Amplicon sequence variants

## References

[1] Zhou W, Sailani MR, Contrepois K, Zhou Y, Ahadi S, Leopold SR (2019). Longitudinal multi-omics of host-microbe dynamics in prediabetes. Nature..

[2] Lloyd-Price J, Arze C, Ananthakrishnan AN, Schirmer M, Avila-Pacheco J, Poon TW (2019). Multi-omics of the gut microbial ecosystem in inflammatory bowel diseases. Nature..

[3] Lim MY, Hong S, Kim BM, Ahn Y, Kim HJ, Nam YD (2020). Changes in microbiome and metabolomic profiles of fecal samples stored with stabilizing solution at room temperature: a pilot study. Sci Rep.

[4] Song SJ, Amir A, Metcalf JL, Amato KR, Xu ZZ, Humphrey G, et al. Preservation methods differ in fecal microbiome stability, affecting suitability for field studies. mSystems. 2016;1(3). 10.1128/mSystems.00021-16. 10.1128/mSystems.00021-16 PMC5069758 27822526

[5] Lim MY, Song EJ, Kim SH, Lee J, Nam YD (2018). Comparison of DNA extraction methods for human gut microbial community profiling. Syst Appl Microbiol.

[6] Santiago A, Panda S, Mengels G, Martinez X, Azpiroz F, Dore J (2014). Processing faecal samples: a step forward for standards in microbial community analysis. BMC Microbiol.

[7] Wagner Mackenzie B, Waite DW, Taylor MW (2015). Evaluating variation in human gut microbiota profiles due to DNA extraction method and inter-subject differences. Front Microbiol.

[8] Jones MB, Highlander SK, Anderson EL, Li W, Dayrit M, Klitgord N (2015). Library preparation methodology can influence genomic and functional predictions in human microbiome research. Proc Natl Acad Sci U S A.

[9] Costea PI, Zeller G, Sunagawa S, Pelletier E, Alberti A, Levenez F (2017). Towards standards for human fecal sample processing in metagenomic studies. Nat Biotechnol.

[10] Sinha R, Abu-Ali G, Vogtmann E, Fodor AA, Ren B, Amir A (2017). Assessment of variation in microbial community amplicon sequencing by the microbiome quality control (MBQC) project consortium. Nat Biotechnol.

[11] Hallmaier-Wacker LK, Lueert S, Roos C, Knauf S (2018). The impact of storage buffer, DNA extraction method, and polymerase on microbial analysis. Sci Rep.

[12] Videnska P, Smerkova K, Zwinsova B, Popovici V, Micenkova L, Sedlar K (2019). Stool sampling and DNA isolation kits affect DNA quality and bacterial composition following 16S rRNA gene sequencing using MiSeq Illumina platform. Sci Rep.

[13] Qiagen: QIAamp PowerFecal Pro DNA Kit Product Profile. 2018. https://www.qiagen.com/ie/resources/resourcedetail?id=5dfb8ed8-32e7-4427-83ba-9a5d2cfc8758&lang=en. Accessed 23 Mar 2020.

[14] de Boer R, Peters R, Gierveld S, Schuurman T, Kooistra-Smid M, Savelkoul P (2010). Improved detection of microbial DNA after bead-beating before DNA isolation. J Microbiol Methods.

[15] Callahan BJ, McMurdie PJ, Rosen MJ, Han AW, Johnson AJ, Holmes SP (2016). DADA2: high-resolution sample inference from Illumina amplicon data. Nat Methods.

[16] Bolyen E, Rideout JR, Dillon MR, Bokulich NA, Abnet CC, Al-Ghalith GA (2019). Reproducible, interactive, scalable and extensible microbiome data science using QIIME 2. Nat Biotechnol.

[17] Bokulich NA, Kaehler BD, Rideout JR, Dillon M, Bolyen E, Knight R (2018). Optimizing taxonomic classification of marker-gene amplicon sequences with QIIME 2's q2-feature-classifier plugin. Microbiome..

[18] DeSantis TZ, Hugenholtz P, Larsen N, Rojas M, Brodie EL, Keller K (2006). Greengenes, a chimera-checked 16S rRNA gene database and workbench compatible with ARB. Appl Environ Microbiol.

[19] Love MI, Huber W, Anders S (2014). Moderated estimation of fold change and dispersion for RNA-seq data with DESeq2. Genome Biol.

