# Supplementary material for: Evaluation of fecal DNA extraction protocols for human gut microbiome studies
Source: BMC Microbiol. 2020 Jul 17;20:212. doi: 10.1186/s12866-020-01894-5 (PMC7367376; doi:10.1186/s12866-020-01894-5)
Supplement: Supplementary file 1 — Additional file 1: Figure S1. A260/A230 of the DNA samples extracted through protocols S, SB, and P. (** P-value < 0.01, *** P-value < 0.001; Kruskal-Wallis test with Dunn’s multiple comparison test). [file 12866_2020_1894_MOESM1_ESM.pdf]

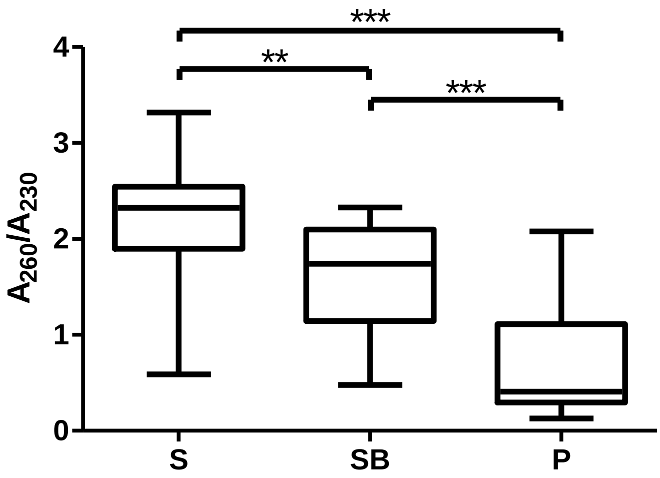

Figure S1. A_{260}/A_{230} of the DNA samples extracted through protocols S, SB, and P. (** P-value < 0.01, *** P-value < 0.001; Kruskal-Wallis test with Dunn's multiple comparison test).
