# Supplementary material for: Evaluation of fecal DNA extraction protocols for human gut microbiome studies
Source: BMC Microbiol. 2020 Jul 17;20:212. doi: 10.1186/s12866-020-01894-5 (PMC7367376; doi:10.1186/s12866-020-01894-5)
Supplement: Supplementary file 2 — Additional file 2: Figure S2. Alpha diversity (observed ASVs and Pielou’s evenness) of the samples extracted through protocols S, SB, and P. (* P-value < 0.05, ** P-value < 0.01; Kruskal-Wallis test with Dunn’s multiple comparison test). [file 12866_2020_1894_MOESM2_ESM.pdf]

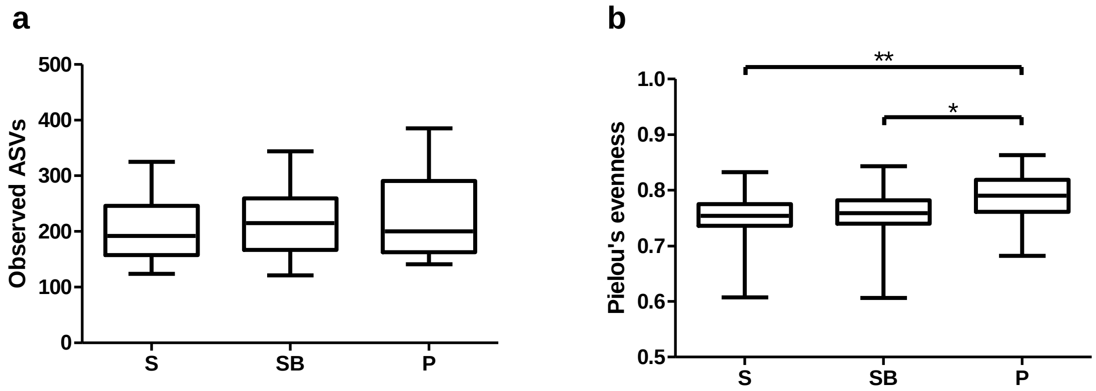

Figure S2. Alpha diversity (observed ASVs and Pielou's evenness) of the samples extracted through protocols S, SB, and P. (* P-value < 0.05, ** P-value < 0.01; Kruskal-Wallis test with Dunn's multiple comparison test).
