# Supplementary material for: Evaluation of fecal DNA extraction protocols for human gut microbiome studies
Source: BMC Microbiol. 2020 Jul 17;20:212. doi: 10.1186/s12866-020-01894-5 (PMC7367376; doi:10.1186/s12866-020-01894-5)
Supplement: Supplementary file 4 — Additional file 4: Figure S3. Average relative abundance plot for DNA extraction protocols (the top 20 most abundant genera). [file 12866_2020_1894_MOESM4_ESM.pdf]

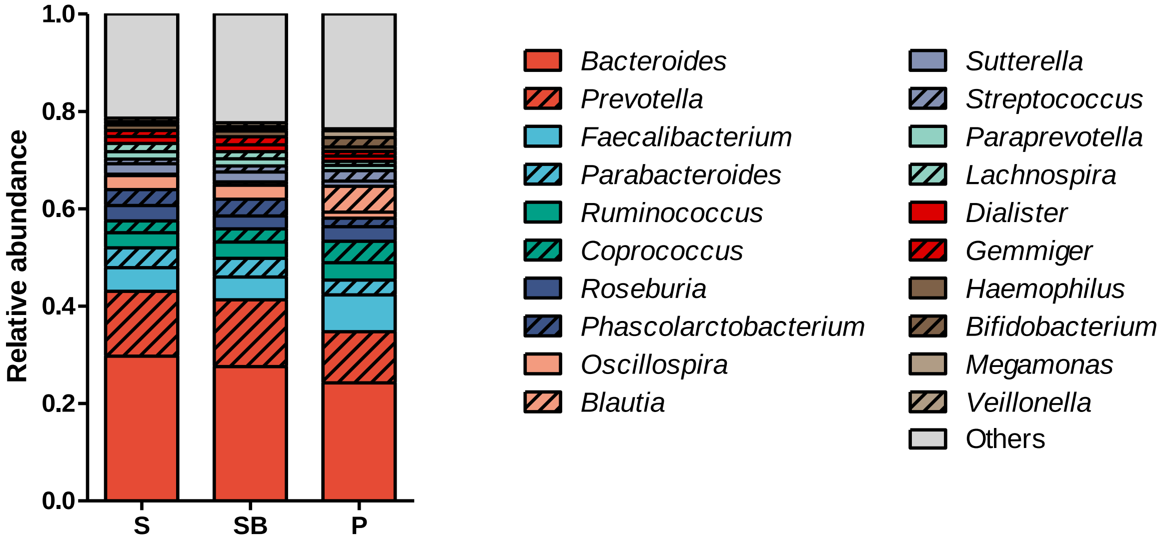

Figure S3. Average relative abundance plot for DNA extraction protocols (the top 20 most abundant genera).
