# Supplementary material for: Evaluation of fecal DNA extraction protocols for human gut microbiome studies
Source: BMC Microbiol. 2020 Jul 17;20:212. doi: 10.1186/s12866-020-01894-5 (PMC7367376; doi:10.1186/s12866-020-01894-5)
Supplement: Supplementary file 5 — Additional file 5: Figure S4. The relative abundances of several significantly differentially abundant genera obtained through protocol P in comparison with protocols SB and S. [file 12866_2020_1894_MOESM5_ESM.pdf]

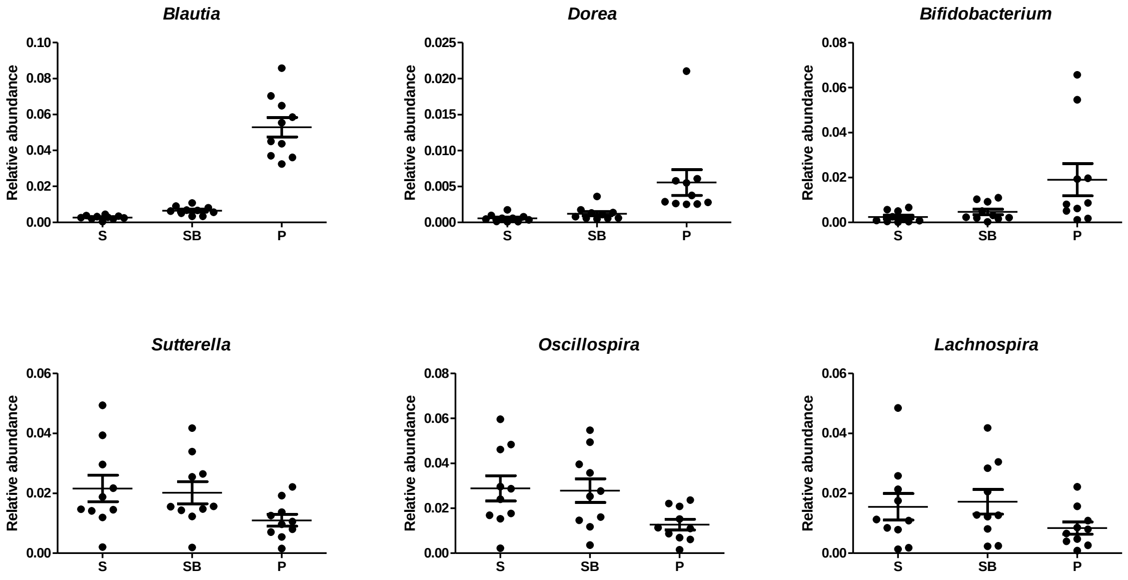

Figure S4. The relative abundances of several significantly differentially abundant genera obtained through protocol P in comparison with protocols SB and S.
